# Diagnosis of Hereditary Pancreatitis Following the Initial Acute Episode With Multiple Pseudocyst Complications

**DOI:** 10.7759/cureus.73653

**Published:** 2024-11-13

**Authors:** Michimasa Fujiwara

**Affiliations:** 1 Pediatrics, National Hospital Organization Fukuyama Medical Center, Fukuyama, JPN

**Keywords:** chronic pancreatitis, genetic testing, hereditary pancreatitis, pancreatic cysts, prss1, pseudocysts

## Abstract

Hereditary pancreatitis (HP) is an unusual form of pancreatitis inherited as an autosomal dominant disorder. Patients typically present with recurrent acute pancreatitis-like symptoms that eventually progress to chronic pancreatitis, resulting in pancreatic exocrine insufficiency or diabetes mellitus, and a high risk of developing pancreatic cancer. As such, early diagnosis is crucial. Herein, we present the case of an 11-year-old boy with no significant medical history, but a family history of type 1 diabetes and pancreatic cancer, who presented with intermittent epigastric pain and nausea. Imaging revealed multiple pancreatic pseudocysts, pancreatic stones, and pancreatic duct dilation, resulting in the diagnosis of acute-on-chronic pancreatitis. Genetic testing confirmed the presence of a mutation in the PRSS1 gene, ultimately resulting in the diagnosis of HP. The patient remained symptom-free for five years during follow-up post-treatment. This case highlights the importance of considering HP in young patients presenting with pseudocysts and other signs of chronic pancreatitis even during the initial acute episode.

## Introduction

Hereditary pancreatitis (HP) is an autosomal dominant condition caused by mutations in the PRSS1, SPINK1, CFTR, and CTRC genes [1]. HP involves genetic and environmental factors that can cause an imbalance in protease regulation, resulting in pancreatic damage. Most patients experience recurrent attacks of acute pancreatitis-like symptoms, such as abdominal pain, nausea, and vomiting, often from around the age of 10 years, which subsequently progress to chronic pancreatitis. Compared to chronic pancreatitis associated with other causative diseases, HP develops at a younger age, and has a longer disease duration, resulting in a higher incidence of pancreatic exocrine insufficiency, diabetes mellitus [2], and pancreatic cancer [3]. As such, the early diagnosis of HP is essential.

Pancreatic pseudocysts arise from inflammatory or obstructive events within the pancreas, resulting in the disruption of the pancreatic duct and leakage of enzyme-rich pancreatic fluid into the retroperitoneum, triggering autodigestion of the surrounding tissue [4,5]. They are less common after acute pancreatitis than after chronic pancreatitis [6]. Furthermore, because they usually take four or more weeks to mature from the onset of acute pancreatitis, they rarely develop during the first attack of an acute pancreatitis-like episode [7]. Herein, we describe a case in which the presence of multiple pseudocysts at the initial onset of an acute pancreatitis-like episode resulted in an early diagnosis of HP.

## Case presentation

**Figure 1:**
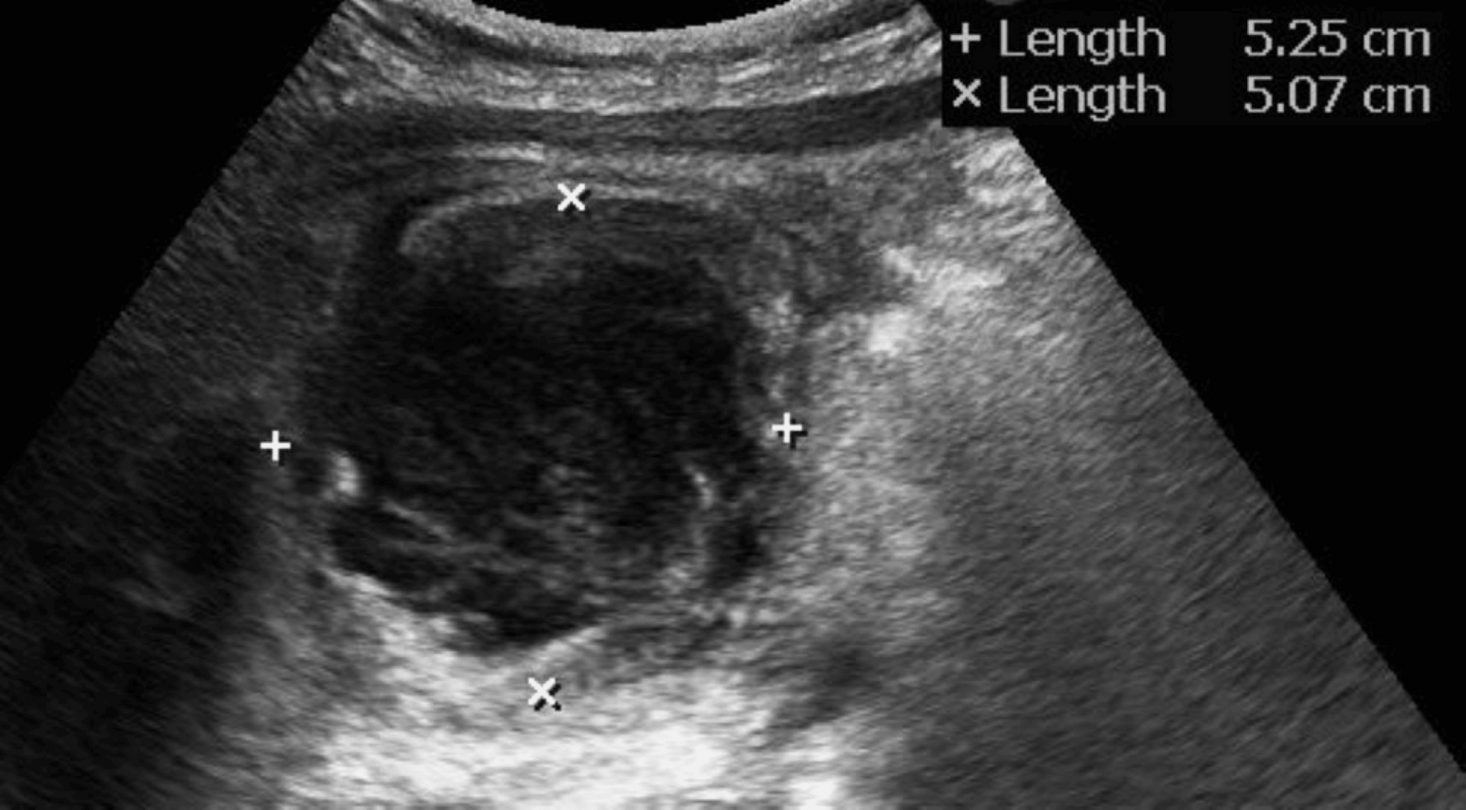
The abdominal US shows a giant cyst 50 mm in diameter on the ventral side of the descending duodenum.

**Figure 2:**
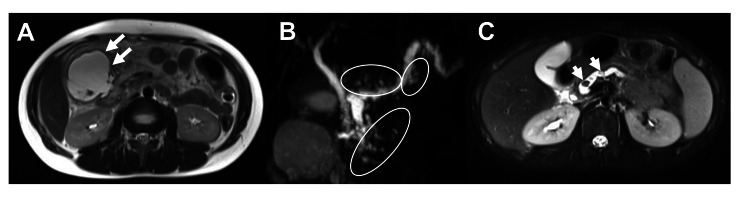
Abdominal MRI and MRCP findings Axial contrast-enhanced T1-weighted MRI (A) shows a round cystic lesion with homogenous fluid density and a well-defined wall (arrows). MRCP (B) shows numerous small cysts on the ventral side of the pancreatic body (circles). T2-weighted MRI (C) shows defects in the main pancreatic duct (arrows), as well as dilatation of the main pancreatic duct and thinning of the pancreatic parenchyma. MRI: magnetic resonance imaging; MRCP: magnetic resonance cholangiopancreatography

An 11-year-old boy presented with intermittent epigastric pain and nausea for one week prior to admission. On the day of admission, he presented with abdominal pain that was difficult to control and was referred to our hospital for subsequent examination and treatment. His medical history was unremarkable; however, he had a family history of type 1 diabetes mellitus in his father, which began at the age of 35 years, as well as pancreatic cancer in his paternal grandfather. His vital signs at the initial visit were as follows: body temperature 38.1°C, heart rate 116 beats per minute, blood pressure 94/71 mmHg, SpO2 97% (room air), and respiratory rate 20 breaths per minute. He was 154.3 cm tall, weighed 45.3 kg, and had a normal body mass index of 19. He experienced a 3 kg weight loss at three months. Physical examination revealed tenderness in the epigastric and right upper quadrants, but no obvious signs of peritoneal irritation. Laboratory investigations further revealed elevated pancreatic enzyme levels. A complete blood count showed increased white blood cell count and neutrophilia. Table 1 presents the detailed laboratory findings. The abdominal US revealed a giant cyst 50 mm in diameter on the ventral side of the descending duodenum (Figure 1), consistent with his right hypochondriac pain. Axial contrast-enhanced T1-weighted magnetic resonance imaging (MRI) of the abdomen revealed a round cystic lesion with homogeneous fluid density and a well-defined wall, consistent with the ultrasound findings (Figure 2A, arrows). Magnetic resonance cholangiopancreatography further revealed numerous small cysts on the ventral side of the pancreatic body, suggestive of pancreatic pseudocysts (Figure 2B, circles). In addition, T2-weighted MRI revealed defects in the primary pancreatic duct indicative of pancreatic stones (Figure 2C, arrows), dilatation of the main pancreatic duct, and thinning of the pancreatic parenchyma suggestive of chronic pancreatitis. Based on these findings, acute-on-chronic pancreatitis was diagnosed. Although the patient’s fever resolved within two days of fluid resuscitation, treatment resulted in an incomplete response. Endoscopic retrograde cholangiopancreatography was subsequently performed to remove the pancreatic stones. However, lithotripsy could not be performed. Therefore, extracorporeal shockwave lithotripsy was performed, following the insertion of a pancreatic stent to remove the stones. The patient was subsequently started on a fat-restricted diet and discharged on the 23rd day of hospitalization. A definitive diagnosis of HP was made following the detection of a PRSS1 (p.G208A/G) mutation in both the patient and his father. Five years after onset, there was no recurrence. The patient is currently undergoing follow-up for diabetes mellitus and pancreatic cancer.

**Table 1:** Laboratory examination of the patient WBC: white blood cell; CRP: C-reactive protein; AST: alanine transaminase; ALT: aspartate transaminase; LD: lactate dehydrogenase; AMY: amylase

Parameters	Patient's values	Reference range
WBC (/μL)	14,800	3,300-8,600
Neutrophils (%)	90.5	38-74
Lymphocytes (%)	5.9	16.5-49.5
Hemoglobin (g/dL)	14.6	13.7-16.8
Platelet count (×10^3^/μL)	366	158-348
CRP (mg/dL)	2.2	<0.14
AST (U/L)	16	13-30
ALT (U/L)	10	10-42
LD (U/L)	265	124-222
AMY (U/L)	638	44-132
Lipase (U/L)	246	13-55
T-bil (mg/dL)	0.3	0.4-1.5

## Discussion

In the present study, we describe a rare case of HP with a PRSS1 mutation diagnosed based on the identification of multiple pseudocysts during the first acute pancreatitis-like episode. An HP may lead to a diagnosis triggered by the presence of pseudocysts. Furthermore, HP should be considered, even at the initial presentation of acute pancreatitis, if there are symptoms such as intermittent abdominal pain and weight loss, or findings such as pseudocysts, pancreatic stones, or dilation of the pancreatic duct indicative of chronic pancreatitis.

Acute recurrent pancreatitis is the most common presentation of HP associated with PRSS1 mutations, but is rarely diagnosed at the initial presentation. The median ages at first onset and diagnosis are 10 and 19 years, respectively [2]. Early diagnosis and intervention are important, because delayed diagnosis is associated with an increased risk of various complications associated with chronic pancreatitis.

The complication rate of pseudocysts in HP varies from 23% [2] to 5.5% [8], depending on the study. Acute pseudocysts complicating acute pancreatitis account for less than 5% of cases [9], and generally develop four to five weeks following the onset of acute episodes [7], with a minimum of two weeks [10]. Pseudocysts are typically absent at the onset of acute pancreatitis-like episodes [11]. Additionally, pseudocysts occur in 20-40% of patients with chronic pancreatitis [9]. Most patients are asymptomatic; however, a broad spectrum of clinical manifestations may develop, depending on the location and extent of fluid accumulation. This was an extremely rare case of an acute pancreatitis-like episode with multiple concurrent pseudocysts at the initial onset of HP. The cause of the pseudocysts was thought to be chronic pancreatitis; however, no symptoms indicative of chronic pancreatitis were observed prior to the onset of the initial symptoms. Therefore, it is difficult to diagnose this disease before its onset. When intra-abdominal cysts are complicated by the initial onset of acute pancreatitis-like episodes, as in the present case, the possibility of HP as an underlying disease should be considered.

HP should also be considered when symptoms or findings suggestive of chronic pancreatitis are observed at the initial presentation of acute pancreatitis. Symptoms of acute pancreatitis include abdominal pain, nausea, and vomiting. Abdominal pain is generally acute, persistent, or severe. The location is the epigastric, as well as the right and left upper quadrants [12,13]. In contrast, symptoms of chronic pancreatitis include abdominal pain, nausea, vomiting, and weight loss. In general, abdominal pain is intermittent and severe. It is located epigastrically and is sometimes poorly localized [12,14]. The present case had both of these features (Table 2). Additionally, pseudocysts, pancreatic stones, and dilation of the pancreatic duct, as in the present case, are common imaging findings in chronic pancreatitis [6,14].

**Table 2:** Clinical symptoms of pancreatitis

	Acute pancreatitis	Chronic pancreatitis	Present case
Abdominal pain	+	+	+
- Character	acute onset, persistent	intermittent or persistent	intermittent
- Intensity	severe	highly variable	moderate
- Location	epigastric	epigastric	epigastric
	right or left upper quadrant	sometimes poorly localized	right upper quadrant
Nausea and/or vomiting	+	+	+
Weight loss	-	+	+

Usually, in patients in their early teens, the coexistence of symptoms and findings of acute and chronic pancreatitis at the initial onset is very rare, with the exception of HP. When symptoms and findings indicate that chronic pancreatitis is complicated at the initial onset of acute pancreatitis-like episodes, as in the present case, the possibility of HP as an underlying disease should be considered. In the present case, multiple findings indicative of chronic pancreatitis, including pseudocysts, were observed at the initial presentation.

## Conclusions

Considering the age at first onset of HP, pediatricians are likely to be involved in the treatment and management of this disease. Early diagnosis and therapeutic intervention are desirable, as persistent pancreatic inflammation may be a risk factor for the development of diabetes mellitus and pancreatic cancer. Overall, the present case indicates that even in the first acute pancreatitis-like episode, when clinical findings suggest chronic pancreatitis, it is necessary to actively collect family history data and consider genetic testing for HP. As such, the results of the present case will aid clinicians in diagnosing similar cases of HP.
